# Anti-CaSR egg yolk antibody improves calcium absorption and bone mineral deposition through intestinal CaSR modulation

**DOI:** 10.1016/j.psj.2026.107824

**Published:** 2026-09-23

**Authors:** Jiakun Yan, Chong Pan, Lirong Fan, Sen Li, Peng Li, Shuangshuang Guo, Yanli Liu, Xiaojun Yang, Binying Ding, Zhouzheng Ren

**Affiliations:** a Hubei Key Laboratory of Animal Nutrition and Feed Science, Wuhan Polytechnic University, Wuhan, Hubei, 430023, China; b College of Animal Science and Technology, Northwest A&F University, Yangling, Shaanxi, 712100, China

**Keywords:** CaSR, Egg yolk antibody, Calcium absorption, Calcium transporters, Bone mineralization

## Abstract

Calcium-sensing receptor (CaSR) is a key negative regulator of intestinal calcium transport, and targeting CaSR represents a promising strategy to improve mineral retention. Egg yolk antibodies can be used as a feed-based approach to modulate intestinal physiological processes. Therefore, this study aimed to evaluate whether anti-CaSR egg yolk antibodies could modulate intestinal calcium transport and enhance calcium and phosphorus retention in broilers. Hy-Line Brown laying hens were immunized over a 4-week period with either a CaSR peptide vaccine or an adjuvant control (without CaSR peptide). High titers of anti-CaSR antibodies were detected in egg yolk at week 5, indicating successful antibody production. The egg yolks were subsequently collected, freeze-dried into powder, and incorporated into broiler diets at a supplementation level of 0.5%. A 14-day feeding trial demonstrated that dietary supplementation with anti-CaSR antibodies markedly increased serum calcium and phosphorus levels, improved apparent ileal digestibility of both minerals, and enhanced tibial ash as well as calcium and phosphorus deposition. Mechanistically, dietary supplementation with anti-CaSR antibodies upregulated components of the transcellular calcium transport pathway, including transient receptor potential vanilloid 5 (TRPV5) and calbindin-D28k (CaBP-D28k), and elevated serum calcitriol level, indicating activation of a vitamin D–dependent regulatory axis. Furthermore, anti-CaSR increased the expression of key phosphate transporters in both the intestine and kidney, supporting its broader role in mineral homeostasis. These findings demonstrate that anti-CaSR antibodies enhance the expression of calcium and phosphorus transport proteins, thereby improving mineral retention and promoting bone mineralization in broilers.

## Introduction

Calcium is a critical nutrient for skeletal development in broiler chickens. The rapid growth of modern broilers places high demands on calcium metabolism, and inadequate calcium utilization contributes to skeletal abnormalities, including tibial dyschondroplasia, leg weakness, and impaired locomotion (David et al., 2023). These disorders not only compromise animal welfare but also cause substantial economic losses in poultry production. Therefore, improving calcium absorption and retention is essential for supporting skeletal development and maintaining broiler health (Li et al., 2017).

Traditionally, calcium nutrition research has focused on the release of soluble calcium from dietary sources, which largely determines bioaccessibility (Walk et al., 2021). However, the intestinal absorption efficiency of conventional calcium supplements, such as calcium carbonate and calcium phosphate, is only 30–50% (David et al., 2019). Simply increasing dietary calcium often does not improve absorption and may even reduce it, due to intestinal feedback mechanisms (Walk et al., 2022). Among these, the calcium-sensing receptor (CaSR), a Class C G-protein coupled receptor, is expressed in the parathyroid glands, kidneys, and gastrointestinal tract, including the basolateral membranes of crypt and villous epithelial cells in the intestine (Magno et al., 2011). CaSR senses luminal calcium and modulates intestinal calcium transport, in part by suppressing vitamin D–stimulated calcium absorption and downregulating TRPV6-mediated calcium uptake via the phospholipase C pathway (Lee et al., 2019; Ling et al., 2021). These findings identify CaSR as a key negative regulator of intestinal calcium transport, suggesting that targeted modulation of CaSR could enhance calcium absorption.

Our laboratory has long focused on developing strategies to target host molecules in the gastrointestinal tract using egg yolk–derived antibodies as a nutritional intervention (Ren,et al., 2019; Ren et al., 2022). Egg yolk antibodies can be generated against specific target proteins through immunization, enabling the production of antigen-specific antibodies in egg yolk with high specificity and binding capacity (Redwan et al., 2021). Owing to their stability in the gastrointestinal environment and compatibility with feed-based delivery, egg yolk antibodies have attracted increasing attention in animal production for applications related to gut health regulation and nutrient retention (Li et al., 2015; Redwan et al., 2021). Given the pivotal role of CaSR in suppressing intestinal calcium transport, we hypothesized that specific anti-CaSR yolk antibody could alleviate CaSR-mediated inhibition and thereby enhance calcium absorption efficiency. To test this hypothesis, antigenic epitopes of chicken CaSR were identified through bioinformatic prediction and structural modeling, followed by peptide immunization to generate high-titer and highly specific anti-CaSR yolk antibody. The present study investigated the effects of dietary anti-CaSR yolk antibody supplementation on calcium and phosphorus metabolism, endocrine regulation, and bone mineralization in broiler chickens.

## Materials and methods

All animal procedures were approved (DK2021005) by College of Animal Science and Technology Animal Care and Use Committee at the Northwest A&F University and were performed in accordance with the guidelines.

### Peptide selection, vaccine preparation and hen immunization

The potential antigenic epitopes of chicken CaSR, which was sourced from National Center for Biotechnology Information (Reference Sequence: XP_416491.6), were identified using methods previously reported (Bobeck et al., 2012). The 3-dimensional structure of human CaSR protein (UniProtKB/Swiss-Prot: P41180.3) was then used to identify the antigenic epitopes that were located on the protein surface (Geng et al., 2016). In total, five 8-amino-acid epitope peptides (internally coded as YP-1, IAADDDYG; YP-2, SQYSDEEE; YP-3, ALKAGQIP; YP-4, TSVETPYM; and YP-5, GIIEGEPT) were selected. Yellow highlighting on the CaSR molecule denotes peptide region used to make a peptide conjugate for stimulation of antibody production in the laying hen (Fig. 1). Peptides were synthesized by GeneScript (Piscatawy, NJ). Five peptide vaccines and a control vaccine were prepared according to lab procedures previously detailed (Ren et al., 2017). 30-week-old Healthy Hy-Line Brown laying hens were used for production of anti-CaSR egg yolk antibody. Five CaSR-derived synthetic peptides (YP1–YP5) were used as immunogens. A glutaraldehyde conjugation procedure as previously described was used to conjugate CaSR peptide to bovine gamma globulin (Sand et al., 2016). Hens were immunized by intramuscular injection of a mixture of antigen and adjuvant into the breast muscle and leg muscle. The first and second immunizations were spaced 14 days apart, followed by a third immunization after another 14-day interval. 7 days post the third immunization, eggs from the immunized hens were collected. Egg yolks were separated from the albumen, lyophilized and stored at 21°C until needed. Enzyme-linked immunosorbent assay (ELISA) were then used to determine the titer of the antibody. All antibody was fed as dried egg yolk powder. After passing quality control tests (Table 1), these eggs were deemed suitable for large-scale production.

**Fig. 1:**
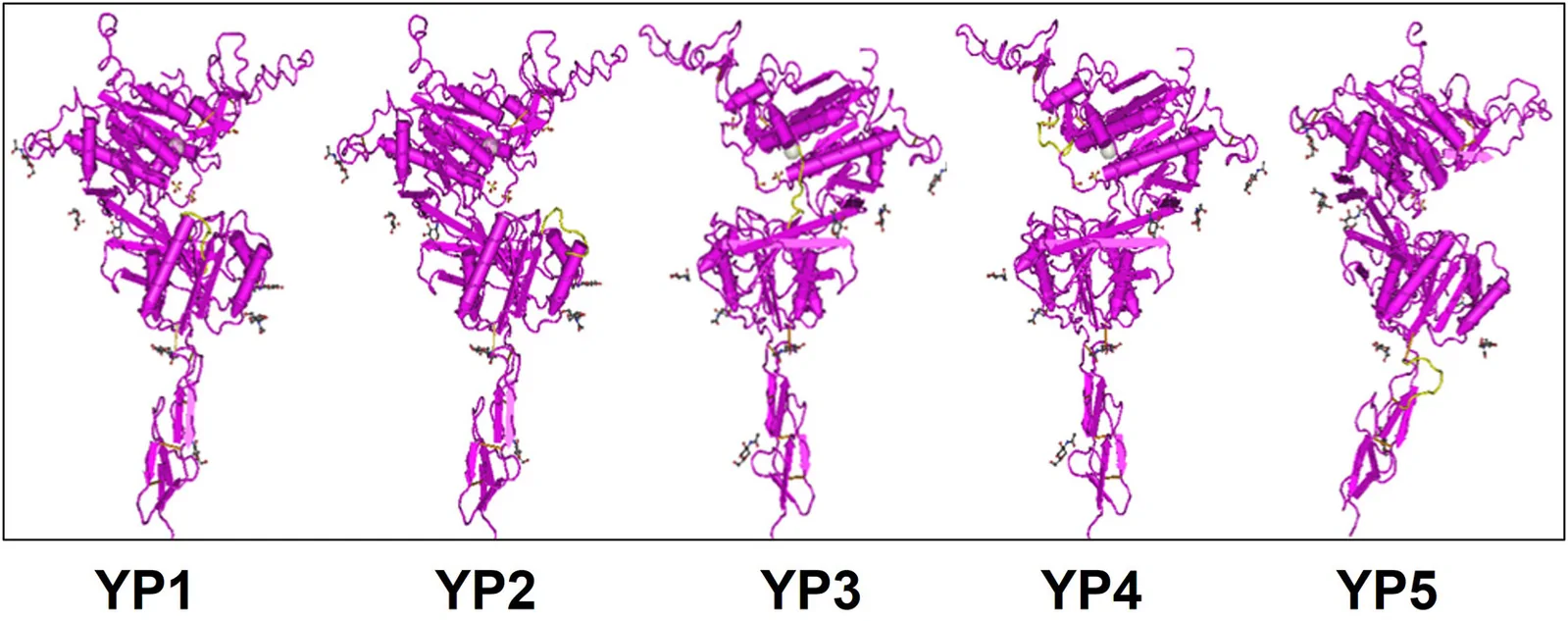
Human CaSR protein structure (UniProtKB/Swiss-Prot: P41180.3) showing the locations of the selected antigenic peptides YP1 (IAADDDYG), YP2 (SQYSDEEE), YP3 (ALKAGQIP), YP4 (TSVETPYM), and YP5 (GIIEGEPT) peptides. Peptide regions used for immunization are highlighted in yellow.

**Table 1:** Reactivity of anti-CaSR yolk IgY against five CaSR-derived peptides at different antibody dilution ratios^a^^,^^b^.

Items		Con on YP	YP on YP	SEM	*P*-value
	YP1	0.256	1.15	0.204	<0.001
	YP2	0.262	1.03	0.179	0.002
1:2000	YP3	0.259	1.09	0.190	<0.001
	YP4	0.245	1.21	0.221	<0.001
	YP5	0.272	1.00	0.166	<0.001
	YP1	0.133	0.982	0.191	0.002
	YP2	0.143	0.775	0.146	0.002
1:4000	YP3	0.151	0.983	0.193	0.002
	YP4	0.170	1.02	0.194	<0.001
	YP5	0.143	0.873	0.165	<0.001
	YP1	0.055	0.804	0.169	<0.001
	YP2	0.057	0.617	0.130	0.002
1:8000	YP3	0.097	0.904	0.182	<0.001
	YP4	0.077	1.00	0.211	<0.001
	YP5	0.063	0.685	0.143	0.012
	YP1	0.035	0.672	0.144	<0.001
	YP2	0.039	0.399	0.084	0.003
1:16000	YP3	0.057	0.691	0.151	0.005
	YP4	0.040	0.740	0.167	0.032
	YP5	0.048	0.527	0.115	0.035

a The significance of the results was evaluated by two-tailed students’ t-tests (n = 3).

b YP1, YP2, YP3, YP4 and YP5 are five 8-amino-acid peptides from calcium-sensing receptor.

### Animals, diets and experimental design

A total of 360 one-day-old male Arbor Acres broiler chickens were used in this study. Birds were randomly assigned to six dietary treatments, with six replicate pens per treatment and ten birds per pen. All broilers were housed under standard environmental conditions and had free access to feed and water throughout the experimental period. The six experimental diets consisted of a basal diet supplemented with 0.5% egg yolk powder, including a control egg yolk powder (CON) and five anti-CaSR egg yolk powders (YP1, YP2, YP3, YP4 and YP5). The feeding trial lasted for 14 d. Diets were formulated to meet the nutritional requirements of broilers according to the Chinese Feeding Standard (NY/T33–2004), and the ingredient composition and nutrient levels are presented in Table S2. At d 14, body weight and residual feed were recorded on a replicate basis. Feed intake, mortality, and body weight (BW) of dead birds were monitored daily during the experiment, and average daily gain (ADG), average daily feed intake (ADFI), feed conversion ratio (FCR), and mortality were calculated for the entire experimental period. After completion of the feeding trial, three birds from each replicate were randomly selected for a 3-d metabolism trial. During this period, total excreta were collected and used for subsequent analyses of nutrient retention.

### Anti-CaSR antibody enzyme-linked immunosorbent assay

The presence and titers of anti-CaSR antibodies were determined using an indirect ELISA based on a previously described method with minor modifications. Briefly, 96-well Nunc MaxiSorp plates (Thermo Fisher Scientific, Rochester, NY, USA) were coated overnight at 4°C with 10 μg/mL of the corresponding CaSR peptide–ovalbuminconjugate diluted in coating buffer (1.59 g/L Na₂CO_3_, 2.93 g/L NaHCO_3_, and 0.2 g/L NaN_3_, pH 9.6). OVA alone was used as the coating antigen for nonspecific binding controls. After coating, plates were blocked with protein-free blocking buffer for 1 h at room temperature and washed three times with PBS containing 0.05% Tween-20.

For egg yolk samples, yolk IgY was extracted by diluting 200 μL of egg yolk with 1.8 mL of acidified PBS (pH 5.0), followed by incubation overnight at 4°C. After centrifugation, the supernatant was collected for antibody detection. Samples were serially diluted in blocking buffer and added to the peptide-OVA-coated plates. Egg yolk samples collected from adjuvant-only immunized hens were used as negative controls. Following incubation, the plates were washed three times with PBST. For chick plasma samples, plasma was appropriately diluted in blocking buffer and analyzed using the same ELISA procedure. For duodenal digesta, 2 g of sample was homogenized with 2 mL of physiological saline and incubated at 37°C for 1 h. The homogenates were subsequently centrifuged at 8,000 × g for 20 min, and the supernatants were collected for antibody detection. The supernatants were appropriately diluted and analyzed using the same indirect ELISA procedure.

After incubation with the samples, goat anti-chicken IgY conjugated with horseradish peroxidase (Diyi Biotechnology, Shanghai, China) was added as the secondary antibody and incubated for 30 min at room temperature. The plates were then washed six times with PBST. The enzymatic reaction was developed by adding substrate solution and incubating for 15 min at room temperature. The reaction was terminated with 0.5 M H₂SO₄, and absorbance was measured at 450 nm using a microplate reader.

### Serum biochemical assay

Blood samples (5 mL, from wing veins) were clotted at 37°C for 60 min in water bath and centrifuged (594 g, 15 min) for serum samples (stored at -80°C). Serum levels of calcium (catalogue no. C004-2), phosphorus (catalogue no. C006-1) and alkaline phosphatase (AKP, catalogue no. A059-2) were analyzed using commercial kits purchased from Nanjing Jiancheng Bioengineering Institute (Nanjing, Jiangsu, China). Serum levels of calcitriol (catalogue no. ml00697414), fibroblast growth factor 23 (FGF23, catalogue no. ml00321112) and parathyroid hormone (PTH, catalogue no. ml00987411) was analyzed using a commercial kit purchased from Shanghai Enzyme-linked Biotechnology Co., Ltd (Shanghai, China). The spectrophotometric reactions were detected using a Synergy HT plate reader (BioTek, Winooski, VT) or a UV-1800 spectrophotometer (Merck; Shimadzu, Japan).

### Determination of calcium and phosphorus contents

Excreta samples were dried to constant weight in a drying oven, equilibrated with ambient air, and milled prior to analysis. Femur samples were oven-dried and subjected to lipid removal. All prepared samples were incinerated in a muffle furnace at 550°C for 6 h, after which the ash was solubilized in hydrochloric acid. Calcium content was quantified by the ethylenediaminetetraacetic acid titration method, whereas phosphorus content was measured using the ammonium vanadate–molybdate colorimetric assay (Nie et al., 2013). Results were expressed on a dry matter basis for excreta and on an ash basis for femur samples.

### Western-blotting analysis

The protein isolation and western blotting procedures were conducted as previously described (Yan et al., 2022). Total protein were extracted and the concentrations were determined with a BCA protein assay kit (catalogue no. WB003, Hat Biotechnology, Xi'an, China). The protein components were electrophoresed in 8 % SDS-PAGE, and transferred electrophoretically to PVDF blotting membranes (catalogue no. 03010040001, Roche Diagnostics, Mannheim, Germany). The membranes were blocked and incubated with the primary antibody (diluted to 1:1000) for 1 h at room temperature and then overnight at 4°C. After three times washing, the secondary antibody (HRP-conjugated, diluted to 1:1000) were applied for 1.5 h. Then, the membranes were washed, probed, and autoradiographed with a Chemiluminescence gel imaging system (DNR, Micro Chemi, Israel) using a Super Signal West Pico Trail Kit (catalogue no. 34580; Pierce, IL). Protein expression levels were quantified by densitometric analysis after normalization to β-actin. Rabbit-derived primary antibodies to type 2a sodium-phosphate co-transporter (NPt2a, catalogue no. A6742), type 2b sodium-phosphate co-transporter (NPt2b, catalogue no. A9460), transient receptor potential vanilloid 5 (TRPV5, catalogue no. A6473), and calbindin-D28k (CaBP-D28k, catalogue no. A0802) were purchased from ABclonal Technology (Wuhan, Hubei, China). A mouse monoclonal antibody against β-actin (catalogue no. CW0096) was purchased from CWBIO Co., Ltd. (Beijing, China). Horseradish peroxidase–conjugated goat anti-rabbit IgG (DY60202; Diyi Biotechnology, Shanghai, China) and goat anti-mouse IgG (bs-0296G-HRP; Bioss Biotechnology, Beijing, China) were used as secondary antibodies.

### Quantitative real-time PCR

The quantitative real‐time RCR analysis was conducted according to previously published procedures (Liu et al., 2018). Total RNA of the samples was extracted using TRIZOL reagent (Invitrogen, Carlsbad, CA) according the manufacturer’s specifications. The concentration and purity of the extracted RNA were determined using a NanoDrop 2000c spectrophotometer (Thermo Scientific, Wilmington, USA). Qualified RNA was subjected to cDNA synthesis using a Primer Script RT Reagent Kit (TaKaRa, Dalian, China). Then, mRNA expression levels of genes were analyzed with a SYBR Premix Ex Taq kit (TaKaRa, Dalian, China) using aniCycler iQ5 real-time PCR machine (Bio-Rad, Hercules, CA). The primers sequences used for Quantitative real-time PCR analysis were listed in Supplemental Table S3. All samples were analyzed in triplicate. Relative gene expression levels were calculated using the chicken β-actin gene as the internal control and determined by the 2^−ΔΔCt^ method.

### Statistical analysis

Data analysis was performed using SPSS version 23.0 (IBM Corp., Chicago, IL). For the determination of anti-CaSR antibody levels in egg yolk and duodenal digesta, comparisons between the CON and YP groups were performed using two-tailed Student’s *t*-tests. For all other measured parameters, data were analyzed using one-way analysis of variance (ANOVA) to evaluate the effects of dietary treatment. When a significant treatment effect was detected, means were separated using Tukey's multiple comparison test. Differences were considered statistically significant at *P* < 0.05. Data are presented as means with pooled SEM.

## Results

### Preparation of anti-CaSR yolk antibody

Immunization of laying hens with CaSR peptides successfully induced the production of yolk antibody with high specificity. As shown in Table 1, egg-derived IgY from hens immunized with CaSR peptides (YP1–YP5) exhibited specific binding to the corresponding antigens, with detectable activity at dilutions up to 1:16,000 (*P* < 0.05), whereas IgY from control hens showed no such reactivity. As shown in Table 2, Vaccination with CaSR peptide YP1, YP2, or YP4 significantly increased serum calcium levels compared with the control group (*P* < 0.05), and there were no significant differences in serum phosphorus levels among the groups (*P* > 0.05). Importantly, no significant differences were observed between the control and immunized groups in BW, daily feed intake, egg weight, laying rate and feed-to-egg ratio (*P* > 0.05).

**Table 2:** Production performance, serum calcium level and serum phosphorus level in laying hens following immunization with CaSR-derived peptides^a^^,^^b^^,^^c^.

Items	CON	YP1	YP2	YP3	YP4	YP5	SEM	*P*-value
Body weight (g)	2109	2058	2070	2098	2148	2126	22.9	0.900
Daily feed intake (g)	136	135	134	138	139	133	2.92	0.510
Average egg weight (g)	61.4	64.2	62.7	66.2	65.7	61.7	0.667	0.164
Laying rate (%)	93.9	96.9	94.9	96.9	93.5	95.4	0.618	0.448
Feed-to-egg ratio	2.35	2.17	2.26	2.16	2.26	2.25	0.022	0.416
Calcium (mmol/L)	1.33^c^	1.43^a^	1.42^a^	1.38^b^	1.39^ab^	1.33^c^	0.011	0.010
Phosphorus (mmol/L)	1.29	1.60	1.62	1.45	1.51	1.38	0.042	0.168

a,b,c means with different superscripts within a row were significantly different (one-way ANOVA, n = 6)

a YP1, YP2, YP3, YP4 and YP5 are five 8-amino-acid peptides from calcium-sensing receptor.

b Body weight, serum calcium level and serum calcium level were measured on week 42.

c Daily feed intake, Average egg weight, Laying rate and Feed-to-egg ratio were assessed from week 32 to week 42.

### Dietary anti-CaSR yolk antibody survives digestion and reaches the duodenum

Compared with the CON group, anti-CaSR yolk antibodies derived from YP1, YP2, YP3, YP4, and YP5 were detectable in the duodenum of broilers fed the supplemented diets. Duodenal digesta samples were collected and analyzed using an indirect ELISA specific for each peptide-derived IgY. As shown in Table 3, All antibody-supplemented groups showed significantly higher optical density (OD₄₅₀) values than the control (*P* < 0.05), with YP3 and YP4 exhibiting the highest signal intensities.

**Table 3:** Binding characteristics of anti-CaSR antibodies in the duodenal chyme with CaSR peptides^a^^,^^b^.

Items	Con on YP	YP on YP	SEM	*P*-value
YP1	0.257	0.671	0.098	0.004
YP2	0.247	0.723	0.113	0.006
YP3	0.207	1.06	0.198	0.002
YP4	0.245	0.860	0.150	0.009
YP5	0.312	0.713	0.100	0.017

a The significance of the results was evaluated by two-tailed students’ t-tests (n = 3).

b YP1, YP2, YP3, YP4 and YP5 are five 8-amino-acid peptides from calcium-sensing receptor.

### Effect of anti-CaSR yolk antibody on serum calcium, phosphorus and hormone levels

As shown in Table 4, one-way ANOVA showed that dietary treatment significantly affected serum phosphorus, calcium, AKP, calcitriol, and FGF23 levels (*P* < 0.05). Specifically, serum calcium level in the YP1, YP2, YP3, and YP4 groups were higher than CON and YP5 groups; serum calcitriol levels in the YP3 and YP4 groups were significantly higher than YP1 groups; compared with the CON and YP1 group, serum FGF23 level were higher in the YP4 groups; but serum AKP level in the YP4 groups were lower than CON and YP1 group. No significant differences in serum PTH level were detected among the groups (*P* > 0.05).

**Table 4:** Effects of anti-CaSR yolk antibody for 14 days on serum phosphorus, calcium, AKP, calcitriol, FGF23, and PTH levels in broilers^a^^,^^b^.

Items	CON	YP1	YP2	YP3	YP4	YP5	SEM	*P*-value
Phosphorus (mmol/L)	2.16	2.27	2.51	2.59	2.60	2.32	0.050	0.030
Calcium (mmol/L)	1.23^b^	1.30^a^	1.31^a^	1.27^a^	1.27^a^	1.21^b^	0.007	<0.001
AKP (mmol/L)	1.59^a^	1.53^a^	1.40^ab^	1.50^ab^	1.32^b^	1.50^ab^	0.024	0.005
Calcitriol (ng/mL)	40.4^ab^	40.0^b^	40.6^ab^	43.0^a^	43.1^a^	41.9^ab^	0.327	0.005
FGF23 (pg/mL)	385^b^	399^b^	416^ab^	412^ab^	442^a^	402^b^	4.55	0.003
PTH (pg/mL)	107	105	107	103	111	112	1.05	0.088

a,b,c means with different superscripts within a row were significantly different (one-way ANOVA, n = 6).

a AKP, alkaline phosphatase; FGF23, fibroblast growth factor 23; PTH, parathyroid hormone.

b YP1, YP2, YP3, YP4 and YP5 are five 8-amino-acid peptides from calcium-sensing receptor.

### Effect of anti-CaSR yolk antibody on tibia development

As shown in Table 5, tibia ash calcium content in the YP5 group was higher than YP2 groups (*P* < 0.05), but no significant differences in tibia-to-body weight ratio, tibia ash content, and tibia ash phosphorus content were detected among the groups (*P* > 0.05). As shown in Fig. 2, serum calcium levels showed a significant positive correlation with tibia-to-body weight ratio (*P* < 0.05), while it was negatively correlated with serum AKP levels (*P* < 0.05). Serum AKP levels showed a significant negative correlation with serum FGF23 levels, tibia ash phosphorus content and tibia ash calcium content (*P* < 0.05). Serum calcitriol levels showed a significant positive correlation with serum FGF23 levels, tibia ash phosphorus content and tibia ash calcium content (*P* < 0.05). Serum FGF23 levels showed a significant positive correlation with tibia ash phosphorus content (*P* < 0.05). tibia ash calcium content showed a significant positive correlation with tibia ash phosphorus content (*P* < 0.05).

**Table 5:** Effects of anti-CaSR yolk antibody for 14 days on tibial mineralization in broilers^a^.

Items	CON	YP1	YP2	YP3	YP4	YP5	SEM	*P*-value
Tibia-to-body weight ratio (%)	0.288	0.315	0.314	0.293	0.302	0.288	0.005	0.323
Tibia ash content (%)	39.1	38.1	38.5	40.4	39.4	37.9	0.320	0.196
Tibia ash calcium content (%)	33.9^ab^	34.1^ab^	33.6^b^	35.2^ab^	35.1^ab^	36.0^a^	0.243	0.020
Tibia ash phosphorus content (%)	17.7	18.0	18.2	18.8	18.4	18.0	0.118	0.126

a,b means with different superscripts within a row were significantly different (one-way ANOVA, n = 6).

a YP1, YP2, YP3, YP4 and YP5 are five 8-amino-acid peptides from calcium-sensing receptor.

**Fig. 2:**
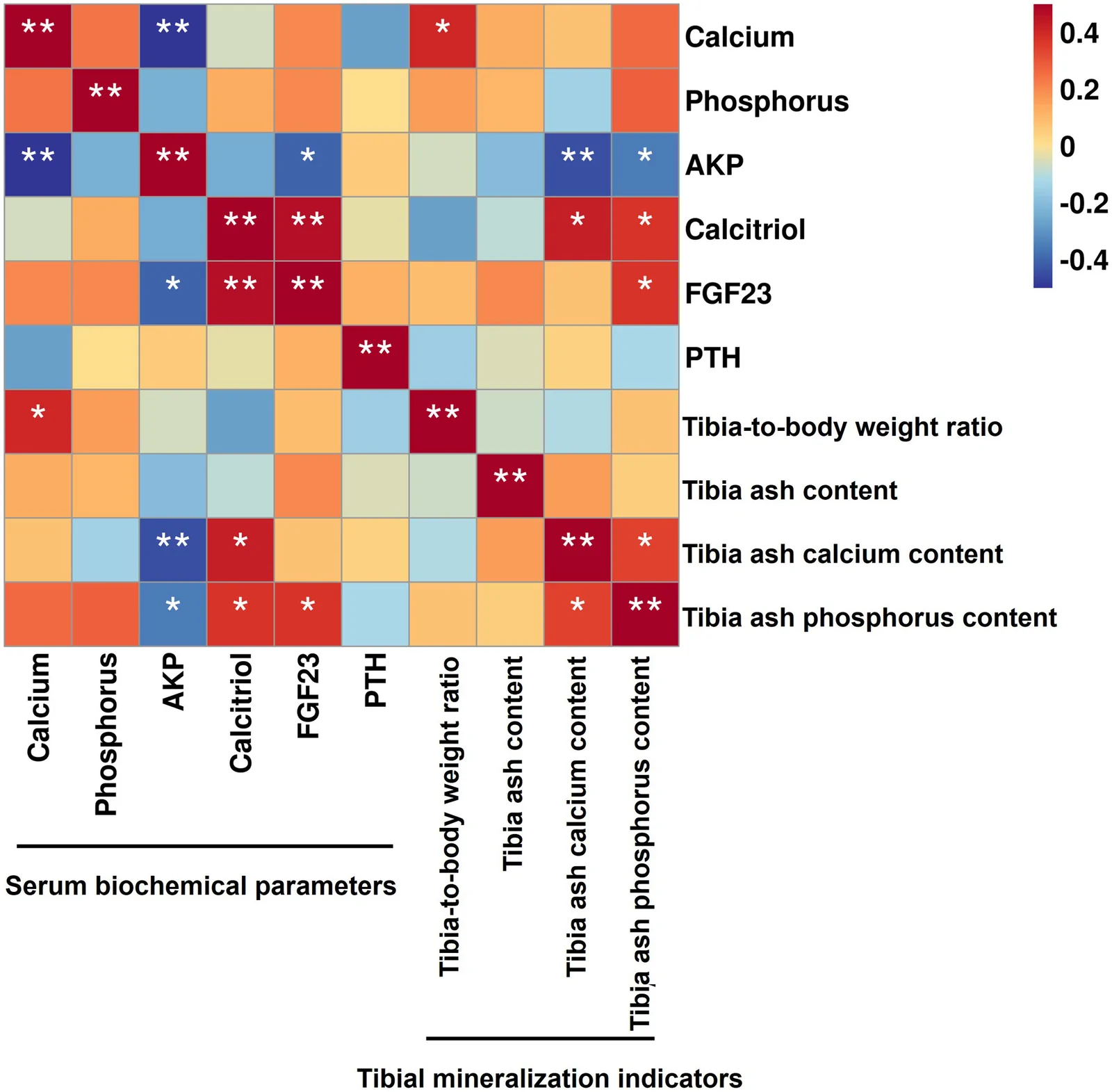
Spearman correlation analysis between serum biochemical parameters and tibial mineralization indicators. AKP, alkaline phosphatase; FGF23, fibroblast growth factor 23; PTH, parathyroid hormone.

### Effect of anti-CaSR yolk antibody on calcium and phosphorus excretion and growth performance

As shown in Table 6, no significant differences in dry excreta calcium level, apparent calcium retention, dry excreta phosphorus level, and apparent phosphorus retention were detected among the groups (*P* > 0.05). As shown in Table S4, no significant changes were observed in growth performance parameters when 0.5% anti-CaSR yolk antibody was supplemented in broiler diets (*P* > 0.05).

**Table 6:** Effects of anti-CaSR yolk antibody for 14 days on tibial mineralization in broilers^a^.

Items	CON	YP1	YP2	YP3	YP4	YP5	SEM	*P*-value
Dry excreta calcium concentration (%)	1.79	1.73	1.61	1.58	1.62	1.69	0.030	0.320
Apparent calcium retention (%)	58.4	61.8	62.8	69.3	64.7	59.0	1.25	0.104
Dry excreta phosphorus concentration (%)	1.36	1.35	1.30	1.36	1.27	1.25	0.018	0.257
Apparent phosphorus retention (%)	58.6	60.9	60.7	63.6	62.1	62.2	0.693	0.424

a YP1, YP2, YP3, YP4 and YP5 are five 8-amino-acid peptides from calcium-sensing receptor.

### Effect of anti-CaSR yolk antibody on renal and duodenal calcium and phosphorus transporter

In the kidney, *SLC34A1* (the gene coding for NPt2a) mRNA expression was significantly higher in the YP2 and YP3 groups than in the CON group (*P* < 0.05; Fig. 3A). *CaBP-D28k* and *TRPV5* mRNA expression were significantly higher in the YP2 and YP3 groups than in the YP4 group (*P* < 0.05; Fig. 3B, 3C). NPt2a protein expression was significantly higher in the YP3 and YP4 groups than in the CON group (*P* < 0.05; Fig. 3D, 3E). No significant differences in CaBP-D28k and TRPV5 protein expression were detected among the groups (*P* > 0.05; Fig. 3F, 3G).

**Fig. 3:**
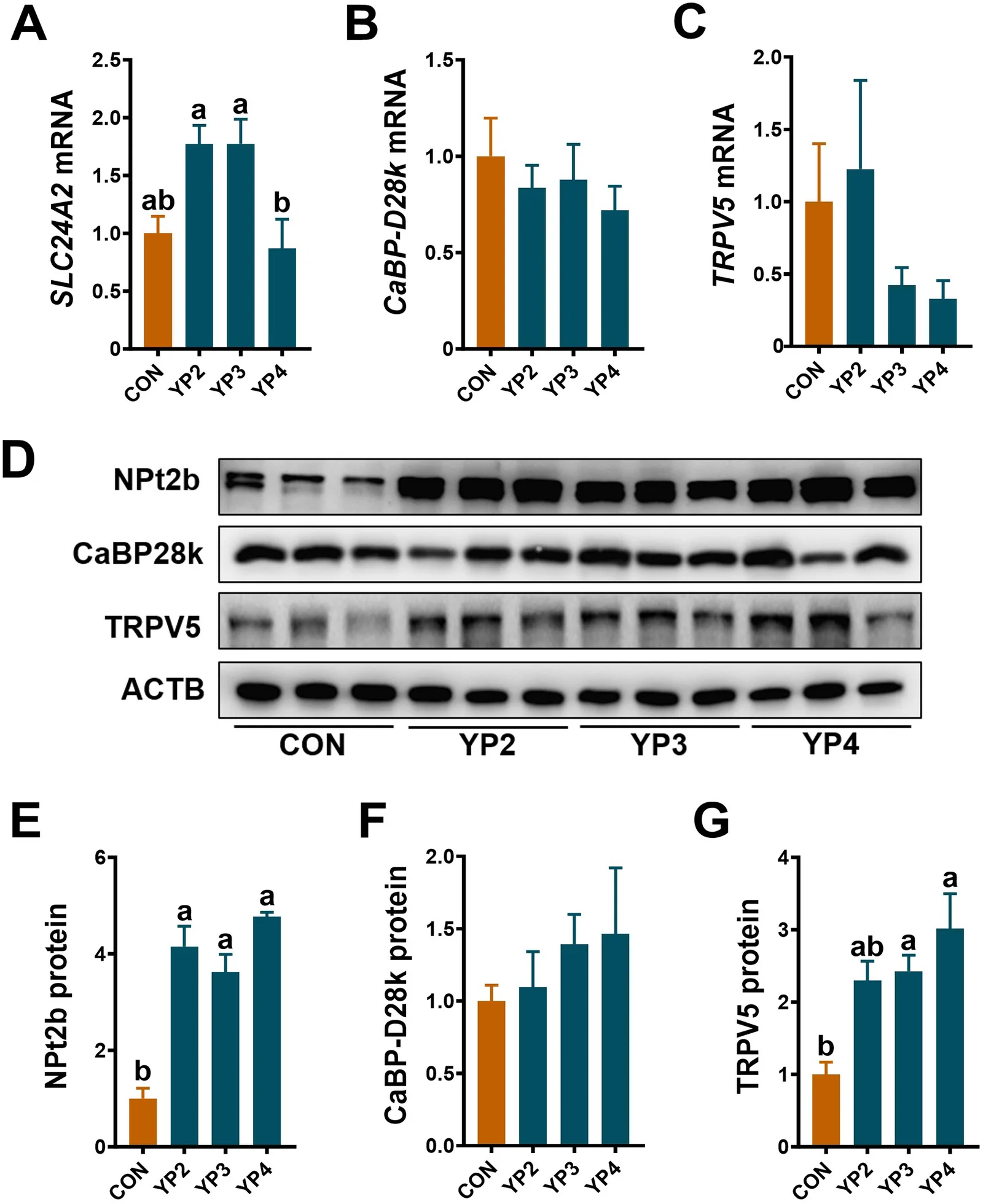
Effects of anti-CaSR yolk antibody for 14 days on duodenal calcium and phosphorus transporters in broilers. (A-C) Relative mRNA levels of *SLC34A2, CaBP-D28k* and *TRPV5* (n = 6), (D-G) Relative protein expression of NPt2b, CaBP-D28k and TRPV5 (n = 3). a-c means with different superscripts among groups were significantly different (one-way ANOVA). *SLC34A2,* the gene coding for NPt2b; NPt2b, type 2b sodium-phosphate co-transporter; CaBP-D28k, calbindin-D28k; TRPV5, transient receptor potential vanilloid 5.

In the Duodenum, *SLC34A2* (the gene coding for NPt2b) mRNA expression was significantly higher in the YP2 and YP3 groups than in the CON group (*P* < 0.05; Fig. 4A). NPt2b and TRPV5 protein expression were significantly higher in the YP2, YP3, and YP4 groups than in the CON group (*P* < 0.05; Fig. 4D, 4E, 4G). No significant differences in *CaBP-D28k* mRNA, *TRPV5* mRNA and TRPV5 protein expression were detected among the groups (*P* > 0.05; Fig. 4B, 4C, 4F).

**Fig. 4:**
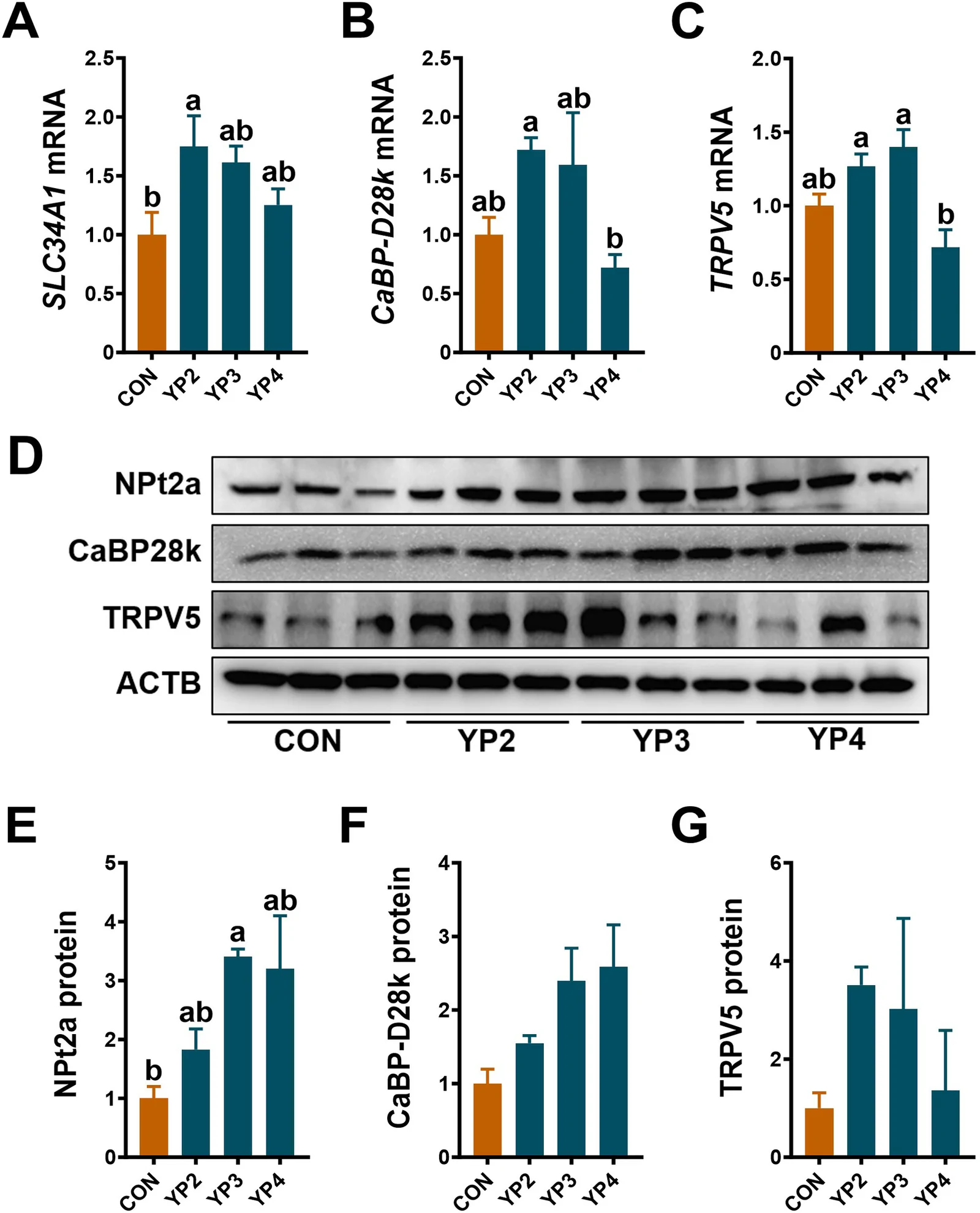
Effects of anti-CaSR yolk antibody for 14 days on renal calcium and phosphorus transporters in broilers. (A-C) Relative mRNA levels of *SLC34A1, CaBP-D28k* and *TRPV5* (n = 6), (D-G) Relative protein expression of NPt2a, CaBP-D28k and TRPV5 (n = 3). a-c means with different superscripts among groups were significantly different (one-way ANOVA). *SLC34A1,* the gene coding for NPt2a; NPt2a, type 2a sodium-phosphate co-transporter; CaBP-D28k, calbindin-D28k; TRPV5, transient receptor potential vanilloid 5.

## Discussion

This study demonstrates that dietary supplementation with anti-CaSR antibody–enriched egg yolk powder represents a promising nutritional strategy for improving calcium and phosphorus retention in broiler chickens. Supplementation with 0.5% egg yolk powder enhanced calcium and phosphorus absorption, reduced mineral excretion, and promoted bone mineralization without negatively affecting growth performance, indicating its efficacy in supporting skeletal development. As IgY-based products are considered safe and can be produced efficiently through egg-derived systems, anti-CaSR yolk antibody offers a practical approach for targeted nutritional modulation of intestinal calcium homeostasis (Wu et al., 2022). These findings highlight the potential application of anti-CaSR IgY–enriched egg yolk powder as a novel feed additive to improve mineral retention and bone health in poultry production.

To develop egg yolk powder with targeted physiological functions, epitope prediction was combined with structural analysis of chicken CaSR (Ling et al., 2021), leading to the identification of five high B-score peptide sequences positioned near calcium-binding sites or interdomain clefts. Immunization with these peptides successfully elicited strong immune responses, generating high titers of specific IgY in yolk, with detectable activity even at a 1:16,000 dilution. These results not only validate the screening strategy as an efficient approach for producing high-affinity antibody but also demonstrate substantial yield potential. Moreover, the production system is scalable, since each immunized hen can provide approximately 40 g of IgY annually without compromising egg production or body weight, indicating its feasibility for sustainable large-scale application (Yakhkeshi et al., 2022). CaSR contains multiple functional sites, and the five antibodies generated in the present study were produced against five different eight-amino-acid regions of CaSR (Ling et al., 2021). Therefore, these antibodies recognize epitopes located at different positions within the extracellular domain of CaSR. Differences in epitope location and the local structural environment may affect antibody accessibility, binding characteristics, and the ability of individual antibodies to alter receptor conformation or CaSR-associated regulatory processes (Fournier et al., 2025). Consequently, antibodies targeting different CaSR epitopes may produce distinct biological responses.

A notable challenge for orally administered antibody is gastric degradation. IgY exhibits relatively high N-glycan content, which confers enhanced stability against proteolysis, acidic conditions, and pepsin digestion (Sheng et al., 2018). In this study, high concentrations of anti-CaSR antibody were detected in the duodenum, suggesting survival through gastric transit and activity in the small intestine. This property is critical for its application as feed additive of intestinal CaSR activity. In most cases, a highly purified form of IgY is not necessary for these products (El-Kafrawy et al., 2023; Yakhkeshi et al., 2022). Interestingly, oral consumption of IgY in egg yolk powder or liquid form has been proven to be more effective than those obtained through extraction and purification. This may be due to the protection from degradation provided by compounds in the yolk (Gu et al., 2021; Ren et al., 2022).

In this study, dietary supplementation with selected anti-CaSR antibody significantly improved indices of mineral metabolism, aligning with enhanced bone mineralization. calcium and phosphorus represent the stoichiometric components of hydroxyapatite, and sufficient circulating pools of these ions are indispensable for bone growth and structural integrity in poultry (Yan et al., 2023). Deficiencies or imbalances in calcium and phosphorus metabolism are well-documented contributors to bone disorders, such as tibial dyschondroplasia and other metabolic bone diseases, highlighting the importance of strategies that promote efficient mineral retention (Landy and Toghyani, 2018). In the present study, anti-CaSR antibody supplementation elevated serum calcium and phosphorus while reducing alkaline phosphatase activity, a biomarker of bone turnover, thereby supporting a shift toward mineral deposition (Nie et al., 2013; Shu et al., 2022). Notably, YP3 and YP4 increased serum calcitriol concentrations, whereas YP2 and YP4 increased FGF23 levels, suggesting differential regulation of endocrine factors involved in Ca/P homeostasis. Consistent with these observations, correlation analysis revealed that serum calcitriol was positively correlated with tibial ash calcium and phosphorus contents, while serum FGF23 was positively correlated with tibial ash phosphorus content. In addition, serum AKP was negatively correlated with both tibial ash calcium and phosphorus contents. These findings collectively highlight the potential of targeted anti-CaSR antibodies to promote bone mineral accrual and reduce the risk of metabolic bone diseases. However, a limitation of the present study is the relatively short 14-day experimental period. As skeletal development and bone mineralization are continuous processes, whether the observed effects of anti-CaSR antibodies persist or change during later growth stages remains unknown. Long-term studies are therefore warranted to evaluate their sustained effects on mineral retention and skeletal development.

PTH, calcitriol, and FGF23 interact in a complex, multi-tissue feedback system that serve to maintain blood calcium and phosphate levels (Blau and Collins, 2015), in which CaSR functions as the calcium sensor calibrating endocrine output (Tan et al., 2021). Previous studies have suggested that CaSR signaling is involved in the regulation of FGF23, and that inhibition of CaSR activity may be associated with reduced circulating FGF23 levels (Daryadel et al., 2024). In contrast, YP2, YP3, and YP4 increased serum FGF23 concentrations in the present study. This apparent discrepancy may be explained by the fact that FGF23 is regulated by multiple physiological factors, including circulating phosphorus and calcitriol, rather than being determined solely by CaSR activity. In the present study, YP2–YP4 also increased serum phosphorus and, in YP3 and YP4, increased calcitriol concentrations. Therefore, the elevation of FGF23 may represent a secondary and compensatory response to changes in systemic mineral metabolism, rather than a direct consequence of CaSR modulation. Increased FGF23 can promote renal phosphorus excretion and suppress excessive calcitriol production, thereby providing a feedback mechanism to prevent excessive elevation of circulating phosphorus and maintain phosphorus homeostasis (Bergwitz and Juppner, 2010). Calcitriol enhances intestinal and renal handling of calcium and phosphate supports matrix mineralization, its increase provides a mechanistic bridge between improved retention and the observed gains in tibial mineral deposition and reduced ALP (Arnold et al., 2021). A possible explanation is that attenuating intestinal CaSR signaling relieves repression of 1α-hydroxylase, thereby favoring conversion of 25-hydroxyvitamin D₃ to calcitriol; this is consistent with reports that reduced CaSR activity augments 1α-hydroxylase and with recognized 1α-hydroxylase expression in intestinal mucosa that can sustain local para/autocrine calcitriol production (Egbuna et al., 2009; Fuchs et al., 2024).

Intestinal absorption and renal reabsorption serve as a central regulator of systemic calcium and phosphorus balance (Arnold et al., 2021), where vitamin D–dependent active transport—mediated by apical TRPV channels and cytosolic calbindin proteins—constitutes the principal pathway for transcellular absorption (Christakos et al., 2016). CaSR is expressed in intestinal epithelial cells and functions as an extracellular Ca²⁺ sensor that negatively regulates active intestinal calcium absorption. In particular, activation of basolateral CaSR has been shown to inhibit TRPV6-mediated transcellular Ca²⁺ transport through a PLC-dependent pathway, providing a local feedback mechanism to prevent excessive calcium absorption (Lee et al., 2019). In our study, dietary anti-CaSR antibody increased duodenal NPt2b and TRPV5, and concomitantly upregulated renal NPt2a, TRPV5, and CaBP-D28k at the transcript and/or protein levels. Orally administered IgY cannot cross the intestinal barrier into the bloodstream (Li et al., 2015). So, these renal effects are unlikely to reflect direct antibody action in the kidney. Rather, they are parsimoniously explained as secondary to endocrine cues, most notably elevated calcitriol, and to greater intestinal mineral availability. Together, the data support a model in which luminal inhibition of intestinal CaSR relieves its negative control over vitamin D signaling, amplifies active calcium and phosphorus transport in the intestine, and entrains coordinated renal reabsorption, which underlies the observed improvements in mineral status and bone outcomes.

Collectively, our findings provide an evidence that orally delivered anti-CaSR antibodies can function as a practical nutritional intervention to enhance calcium and phosphorus retention in poultry. In this study, dietary supplementation with anti-CaSR antibody numerically reduced fecal mineral excretion and improved mineral absorption efficiency, although these differences did not reach statistical significance. These findings suggest that anti-CaSR antibody may enhance mineral bioavailability without requiring additional dietary calcium or phosphorus supplementation, highlighting its potential as a nutritional strategy for improving mineral-use efficiency and promoting more sustainable mineral management. This is particularly significant given that simply elevating dietary mineral inputs often fails to translate into effective utilization by the host (David et al., 2023; Zhang et al., 2025). Moreover, by reducing phosphorus excretion, this strategy may offer an additional benefit in terms of environmental sustainability, mitigating nutrient pollution associated with intensive livestock production (Zhao et al., 2025). A limitation of the present study is that the direct interaction between orally administered anti-CaSR antibody and intestinal CaSR was not demonstrated in vivo, and downstream CaSR signaling activity was not directly assessed. Therefore, the observed changes in mineral metabolism and Ca/P transport-related proteins provide indirect evidence consistent with CaSR-associated regulation, rather than definitive evidence of direct CaSR inhibition by anti-CaSR antibody. Future studies using direct receptor-binding assays and evaluation of CaSR downstream signaling pathways will be necessary to further validate this proposed mechanism.

## Conclusion

In conclusion, this study demonstrates that egg yolk–derived anti-CaSR antibody represents an effective and food-compatible nutritional strategy to enhance calcium and phosphorus retention in broiler chickens. By orally modulating intestinal CaSR activity, anti-CaSR antibody improved mineral absorption efficiency, reduced fecal mineral losses, and promoted bone mineral deposition without negatively affecting growth performance. Mechanistically, these benefits were associated with activation of vitamin D–dependent regulatory pathways and coordinated upregulation of key calcium and phosphate transporters in the intestine and kidney. Among the five peptide-derived antibodies, YP3 and YP4 showed the most favorable overall responses and therefore represent promising candidates for further development as functional feed additives.

## Author contributions

Jiakun Yan designed and conducted the study, coordinated the animal trial, collected and analyzed the data, interpreted the results, and drafted the manuscript. Chong Pan coordinated the animal trial, collected and analyzed the data, interpreted the results. Lirong Fan and Sen Li contributed to the conduct of the study, sample collection. Peng Li, Shuangshuang Guo, Yanli Liu, and Xiaojun Yang contributed to data organization and interpretation, participated in the discussion of the results, and critically reviewed the manuscript. Binying Ding and Zhouzheng Ren conceived and supervised the project, contributed to the study design and interpretation of the findings. All authors read and approved the final manuscript.

## Ethics declaration

This study was conducted in accordance with the following guidelines for animal welfare and/or reporting: National Institute of Animal Health. This study was approved by the College of Animal Science and Technology Animal Care and Use Committee at the Northwest A&F University. (Approval No. DK2021005).

## Disclosures

The authors declared that they have no conflicts of interest to this work. We declare that we do not have any commercial or associative interest that represents a conflict of interest in connection with the work submitted.

## Footnotes

Supplementary material associated with this article can be found, in the online version, at doi:10.1016/j.psj.2026.107824.
