# Supplementary material for: Anti-CaSR egg yolk antibody improves calcium absorption and bone mineral deposition through intestinal CaSR modulation
Source: Poult Sci. 2026 Sep 23;105(12):107824. doi: 10.1016/j.psj.2026.107824 (PMC13659755; doi:10.1016/j.psj.2026.107824)
Supplement: Supplementary file 1 [file mmc1.docx]

**Anti-CaSR egg yolk antibody improves calcium absorption and bone mineral deposition through intestinal CaSR modulation**

Jiakun Yan ^a, b^, Chong Pan ^b^, Lirong Fan ^a^, Sen Li ^b^, Peng Li ^a^, Shuangshuang Guo ^a^, Yanli Liu ^b^, Xiaojun Yang ^b^, Binying Ding ^a^, and Zhouzheng Ren ^b, *^

^a^ Hubei Key Laboratory of Animal Nutrition and Feed Science, Wuhan Polytechnic University, Wuhan, Hubei 430023, China

^b^ College of Animal Science and Technology, Northwest A&F University, Yangling, Shaanxi 712100, China

*** Corresponding author**

poultryren@nwafu.edu.cn (Z Z. Ren); Tel: 86-18191256330

**Table S1.** Composition and nutrient concentrations of basal diet for laying hens

| Ingredients | % |
| --- | --- |
| Corn | 56.69 |
| Soybean meal | 25.77 |
| Distillers dried grains with solubles | 4.00 |
| Calcium carbonate | 9.04 |
| Dicalcium phosphate | 1.15 |
| Soybean oil | 1.51 |
| Sodium chloride | 0.26 |
| DL-Methionine | 0.18 |
| Choline chloride | 0.15 |
| Montmorillonite | 0.25 |
| Premix^1^ | 1 |
| In total | 100.00 |
| Nutrient levels |  |
| Metabolizable energy, kcal/kg (calculated) | 2,600 |
| Crude protein (calculated/analyzed) | 16.5/16.9 |
| Total phosphorus (calculated/analyzed) | 0.53/0.48 |
| Calcium (calculated/analyzed) | 3.50/3.45 |

^1^Provided per kilogram of diet: copper 8 mg, iron 60 mg, zinc 80 mg, manganese 60 mg, selenium 0.3 mg, iodine 0.35 mg, vitamin A 8000 IU, vitamin D_3_ 1600 IU, vitamin E 5 mg, vitamin K_3_ 0.5 mg, thiamine 0.8 mg, riboflavin 2.5 mg, pantothenic acid 2.2 mg, niacinamide 20 mg, pyridoxine 3 mg, biotin 0.1 mg, folic acid 0.25 mg.

**Table S2.** Composition and nutrient concentrations of basal diet for broilers

| Ingredients | % |
| --- | --- |
| Corn | 58.70 |
| Soybean meal | 26.20 |
| Cottonseed meal | 3.00 |
| Corn gluten meal | 5.00 |
| Methionine | 0.17 |
| Threonine | 0.12 |
| Lysine salt | 0.70 |
| Soybean oil | 1.68 |
| Stone powder | 1.41 |
| Dicalcium phosphate | 1.92 |
| Choline chloride | 0.10 |
| Sodium chloride | 0.30 |
| Premix^1^ | 0.20 |
| Yolk powder | 0.50 |
| In total | 100 |
| Nutrient levels |  |
| Metabolizable energy, kcal/kg (calculated) | 2,880 |
| Crude protein (calculated/analyzed) | 21.5/21.8 |
| Total phosphorus (calculated/analyzed) | 0.69/0.68 |
| Calcium (calculated/analyzed) | 1.00/0.93 |

^1^Premix is provided for every kilogram of full price material: copper 8 mg, iron 100 mg, zinc 100 mg, manganese 120 mg, iodine 0.70 mg, selenium 0.30 mg, vitamin A 8000 IU, vitamin D_3_ 1000 IU, vitamin K_3_ 0.5 mg, vitamin E 20 mg, thiamine 2 mg, riboflavin 8 mg, pantothenic acid 10 mg, niacin 35 mg, pyridoxine 3.5 mg, biotin 0.18 mg, folic acid 0.55 mg, vitamin B_12_ 0.01 mg.

**Table S3.** Sequences of primers used for the quantitative real-time PCR analysis^1^

| Gene | Accession no. | Sequence of Primers(from 5’ to 3’) |
| --- | --- | --- |
| *SLC34A2* | NM_204474.2 | F: AAAGTGACGTGGACCATG  R: GAGACCGATGGCAAGATCAG |
| *SLC34A1* | XM_015293846.2 | F: CCAAACTGCACGGCTTCT  R: GGGAGGTCAGTGTTGATGA |
| *TRPV5* | XM_004938143.2 | F: TGGAACGGACTAAGTCAGAAGTTG  R: CGTTATGGCTGGGATGTTGTT |
| *CaBP-D28k* | NM_205513.2 | F: CAGGGTGTCAAAATGTGTGC  R: GCCAGTTCTGCTCGGTAAAG |
| *β-actin* | NM_205518.2 | F: AATCAAGATCATTGCCCCACCT  R: TGGGTGTTGGTAACAGTCCG |

^1^*SLC34A1*, the gene coding for type 2a sodium-phosphate co-transporters (NPt2a); *SLC34A2*, the gene coding for type 2b sodium-phosphate co-transporters (NPt2b); *TRPV5,* transient receptor potential vanilloid 5; *CaBP-D28k,* calbindin-D28k.

**Table S4.** Effects of dietary anti-CaSR antibody on growth performance in broilers^1,2,3^

| Items | CON | YP1 | YP2 | YP3 | YP4 | YP5 | SEM | *P*-value |
| --- | --- | --- | --- | --- | --- | --- | --- | --- |
| Body weight (g) | 503 | 502 | 492 | 498 | 490 | 495 | 3.17 | 0.826 |
| Daily feed intake (g) | 43.5 | 43.8 | 42.2 | 43.7 | 42.6 | 43.6 | 0.278 | 0.509 |
| Average daily gain (g) | 32.9 | 32.8 | 32.1 | 32.5 | 32.1 | 32.7 | 0.238 | 0.889 |
| Feed conversion ratio | 1.32 | 1.33 | 1.32 | 1.34 | 1.33 | 1.35 | 0.005 | 0.257 |

^1^ Body weight was measured on day 14.

^2^ Daily feed intake, Average daily gain, and Feed conversion ratio were assessed from day 1 to day 14.

^3^ YP1, YP2, YP3, YP4 and YP5 are five 8-amino-acid peptides from calcium-sensing receptor.
